# Challenges in the Management of Atrial Fibrillation With Subclinical Hyperthyroidism

**DOI:** 10.3389/fendo.2021.795492

**Published:** 2022-01-04

**Authors:** Baris Gencer, Anne R. Cappola, Nicolas Rodondi, Tinh-Hai Collet

**Affiliations:** ^1^ Division of Cardiology, Department of Medicine, Geneva University Hospitals, Geneva, Switzerland; ^2^ Institute of Primary Health Care (BIHAM), University of Bern, Bern, Switzerland; ^3^ Division of Endocrinology, Diabetes, and Metabolism, Perelman School of Medicine at the University of Pennsylvania, Philadelphia, PA, United States; ^4^ Department of General Internal Medicine, Inselspital, Bern University Hospital, University of Bern, Bern, Switzerland; ^5^ Division of Endocrinology, Diabetology, Nutrition and Therapeutic Education, Department of Medicine, Geneva University Hospitals, Geneva, Switzerland

**Keywords:** atrial fibrillation, subclinical hyperthyroidism, rate control, rhythm control, amiodarone

## Abstract

Subclinical thyroid disorders have a high prevalence among older individuals and women. Subclinical hypothyroidism is diagnosed by elevated serum levels of thyroid-stimulating hormone (TSH) with thyroxine levels within the reference range, and subclinical hyperthyroidism is diagnosed by low TSH in conjunction with thyroxine and triiodothyronine levels within reference ranges. Atrial fibrillation is the most commonly diagnosed cardiac arrhythmia and has been associated with an increased risk of mortality, heart failure, stroke, and depression. Mechanistic data from animal and human physiology studies as well as observational data in humans support an association of subclinical hyperthyroidism with atrial fibrillation. Guidelines recommend the measurement of TSH in the evaluation of new-onset atrial fibrillation. All patients with overt hyperthyroidism should be treated, and treatment of subclinical hyperthyroidism should be considered in patients older than 65 years with TSH < 0.4 mlU/L, or in younger patients with TSH < 0.1 mlU/L. Guidelines also recommend screening for AF in patients with known hyperthyroidism. Wearable devices that measure the heart electrical activity continuously may be a novel strategy to detect atrial fibrillation in patients at risk. In this review, we explore the interplay between thyroid hormones and atrial fibrillation, management controversies in subclinical hyperthyroidism, and potential strategies to improve the management of atrial fibrillation in patients with subclinical hyperthyroidism.

## 1 Introduction

Subclinical thyroid dysfunction (SCTD) is a common condition in the general population, especially in older individuals and women, with a prevalence of 10-15% in some cohort studies (1–3). The prevalence of asymptomatic patients with SCTD is estimated to be 10-15% depending on the cohort (2, 3), while symptoms were more often reported by patients with hypothyroidism than those in euthyroidism although no specific symptom was sensitive enough for diagnosis (2). SCTD comprises both subclinical hypothyroidism and subclinical hyperthyroidism, which are diagnosed using serum blood tests. Subclinical hypothyroidism is diagnosed by elevated serum levels of thyroid-stimulating hormone (TSH) with free thyroxine (T4) levels within the reference range, and subclinical hyperthyroidism is diagnosed by low TSH in conjunction with free T4 and triiodothyronine (T3) levels within reference ranges (4–6).

Most patients with SCTD are asymptomatic or have non-specific symptoms (e.g., fatigue, weight loss/gain, heat/cold intolerance, poor concentration reported as ‘brain fog’). In subclinical hypothyroidism, bradycardia and diastolic hypertension may be found, whereas subclinical hyperthyroidism may be associated with tachycardia and dyspnea on exertion. In addition to increased risk of coronary heart disease in subclinical hypothyroidism (7) and subclinical hyperthyroidism (8), there is an increased risk for arrhythmia in SCTD due to the effects of circulating thyroid hormones on cardiac function. In particular, the risk of atrial fibrillation (AF) ranged from a hazard ratio (HR) of 1.68 (95% confidence interval [CI], 1.16-2.43) (8) to a relative risk (RR) of 3.1 (95% CI 1.7-5.5) (9), depending on the studied population as detailed in section *Association Between Thyroid Function and Atrial Fibrillation*.

In this narrative review, we focus on the latest scientific knowledge on the association between subclinical hyperthyroidism and atrial fibrillation (AF), the most common arrhythmia (10). We also discuss potential strategies to improve the management of AF in patients diagnosed with these two conditions.

## 2 Epidemiology of Atrial Fibrillation and Risk Factors

AF is a common medical condition that increases with age (10). The definition includes paroxysmal, persisting, long-standing and permanent AF based upon the duration of the arrhythmia, and the strategy chosen for cardioversion (11). The lifetime risk for AF is 30-40%, i.e. approximately 1 in 3 individuals will develop this arrhythmia, with an estimated peak of 15 million cases in Europe in 2050 (12). The risk factors contributing to incident AF are usually multifactorial and include heart failure, valvular disease, coronary artery disease, vascular disease, established or borderline hypertension, diabetes, chronic kidney disease, physical inactivity, alcohol consumption, smoking, obesity, inflammatory disease, chronic obstructive pulmonary disease, obstructive sleep apnea, acute illness, surgery, and hyperthyroidism (11). It is recommended to diagnose and treat these risk factors to reduce the burden of AF and its complications. Hyperthyroidism or subclinical hyperthyroidism with low TSH (<0.45 mlU/L) is a reversible, treatable but also an uncommon contributing factor to AF. In any case, guidelines recommend the screening of thyroid disorder in patients with AF.

In addition to these modifiable risk factors, non-modifiable risk factors contributing to the development of AF include ageing, genetics, ethnicity and male sex (13).

AF varies widely in presentation, from asymptomatic or silent AF, to symptomatic AF with palpitations, dyspnea, or fatigue, or, rarely, hemodynamic instability (e.g., syncope, lightheadedness, pulmonary edema, myocardial ischemia, or cardiogenic shock) (11). A diagnosis of AF is associated with a 2-3 fold higher risk of death, as well as stroke, left ventricular dysfunction or heart failure, cognitive decline or vascular dementia, depression, impaired quality of life, and hospitalizations (11).

Integrating the multiple contributing comorbidities is important when managing patients with both AF and SCTD. Indeed, the risk of AF associated with subclinical hyperthyroidism is not a homogenous entity and depends on the co-existence of other risk factors. In a large registry of 40,628 patients with subclinical hyperthyroidism, the male sex, age, coronary heart disease, heart failure and valve disease were all independent predictors of AF (14). All those factors need to be considered in the management of AF. For instance, the reparation of a severe mitral regurgitation is recommended in patients with a dilated left atrium to correct a structural substrate of the arrhythmia (15). Coronary revascularization could also be required in case of acute coronary syndrome or a significant cardiac ischemia.

## 3 Effects of Thyroid Hormones on Heart Rhythm and Function

Thyroid hormones have direct actions on the cardiac and vascular function. In animal models, elevated thyroid hormones act on the β1-adrenergic and muscarinic receptors of the heart resulting in an increased sympathetic function and decreased atrial refractory period. The thyroid hormones also act on the gene expression of major ionic channels with decreased L-type calcium channel, and increased expression of Kv1.5 resulting in shorter action potential duration in left atrium (16, 17). Another animal study reported an increased triggered activity and automaticity located in the pulmonary vein cardiomyocytes with thyroid hormones (18) explaining the potential arrhythmogenic effect of hyperthyroidism in AF *via* decreased action potential duration, increased spontaneous activity in pulmonary veins, increased delayed after-depolarizations in pulmonary veins and increased after-depolarizations.

Thyroid hormones accelerate myocardial inotropy and heart rate, and consequently the cardiac output through two mechanistic pathways: nuclear thyroid receptors and the adrenergic system. The binding of thyroid hormones to nuclear receptors enhances the gene expression of the cardiac myocyte proteins and upregulates sarcoplasmic calcium ATPase, myosin heavy chain alpha, voltage gated K+ channels, Na+ channels and beta1-adrenergic receptors. The mechanisms explaining the increased risks of AF with subclinical hyperthyroidism are not entirely clear, but some possible pathways involve T3: (1) T3 reduces heart rate variability due to the inhibition of the vagal system and results in arrhythmogenic effect; (2) T3 binds to nuclear receptors and increases specific cardiac gene expression; and (3) T3 causes peripheral vasodilatation and interfere with cardiac preload and contractility (9, 19). In addition, metabolites of thyroid hormones, such as diiodothyronine (T2), have been implicated in the process of AF (20). Animal studies have shown the effects of thyroid hormones on conduction in atrial cardiomyocytes, with increased spontaneous activity in the pulmonary veins, shorter duration of action potentials, faster beating rates and higher incidence of delays after depolarization (18).

## 4 Association Between Thyroid Function and Atrial Fibrillation

The increased automaticity and enhanced triggered activity could explain the increased risk of AF observed with subclinical hyperthyroidism (see section *Epidemiology of Atrial Fibrillation and Risk Factors*). Observational studies suggest up to a 2.8-fold increased risk of AF among patients with subclinical hyperthyroidism compared to patients with euthyroidism (8, 9, 21). In a cross-sectional study of 132,707 patients, hyperthyroidism was significantly associated with higher heart rate and prolonged QTc interval (22). Therefore, clinical guidelines recommend testing thyroid hormones in the diagnostic work-up and follow-up of all patients with AF.

Besides subclinical hyperthyroidism, some data investigated the association between free T3 levels and the recurrence of AF after catheter ablation (23). In a cohort of 1115 patients who underwent catheter ablation, those in the lowest quintile (HR 1.60, 95% CI 1.26-2.03), as well as those in the highest quintile of free T3 (HR 1.47, 95%CI 1.16-1.87) had increased risks of AF over a median follow-up of 2 years compared to those in the median quintiles. This U-shaped found with free T3 was not observed with TSH or free T4 and needs to be confirmed in other studies.

The management of SCTD has been debated for many years. All human studies have been observational studies, or small and physiological studies, thus large enough randomized controlled trials are needed (24). Of note, the randomized controlled trial on the incidence of AF after radioiodine treatment of subclinical hyperthyroidism vs. active medical surveillance conducted in France could not recruit enough participants (ClinicalTrials.gov registration noNCT00213720).

**Figure 1:**
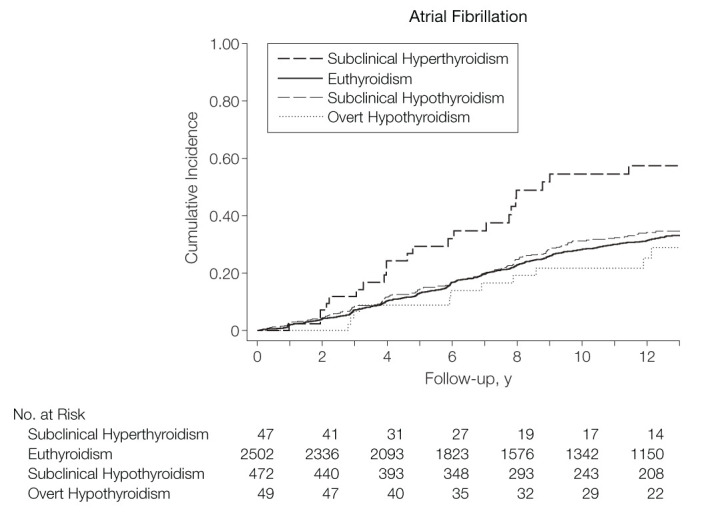
Cumulative incidence of atrial fibrillation according to the thyroid function tests, defined as: subclinical hyperthyroidism TSH 0.10 to 0.44 mIU/L and normal free T4; euthyroidism TSH 0.45 to 4.50 mIU/L; subclinical hypothyroidism TSH > 4.50 and < 20.0 mIU/L, and normal free T4. The 2 individuals with TSH < 0.10 mIU/L were not represented due to the small group size, adapted from Cappola et al. (26) (*with permission to reproduce*).

The advent of the second-generation TSH assay in the 1980s allowed for improved precision of measurement of TSH in the lower ranges. In people aged 60 years and older enrolled in the Framingham Heart Study, Sawin et al. reported an incidence of AF at 28 per 1000 person-years in individuals with TSH ≤ 0.1 mIU/L compared to 16 per 1000 person-years with TSH 0.1 to 0.4 mIU/L, and 11 per 1000 person-years with TSH 0.4 to 5.0 mIU/L (9). The RR of AF in hyperthyroidism was 3.8 (95% 1.7–8.3, P < 0.001) for those with TSH ≤ 0.1 mIU/L and 1.6 (95%CI 1.0–2.5, P = 0.05) for those with TSH 0.1–0.4 mIU/L compared to the euthyroid state. In a subgroup analysis excluding those with thyroid hormone replacement, the RR was slightly more elevated suggesting a higher risk of AF with endogenous hyperthyroidism compared to thyroid overreplacement. In a cross-sectional study from Tenerz et al., AF was present in 28% of patients (mean age 65) with subclinical hyperthyroidism compared to 10% of those who were euthyroid (25). In the Cardiovascular Health Study, Cappola et al. found a greater incidence of AF in subclinical hyperthyroidism than in the euthyroid state over 13 years of follow-up: 67 vs. 31 events per 1000 person-years, respectively (P < 0.001), and a hazard ratio of 2.18 (95%CI 1.42–3.33) (**Figure 1**) (26). Compared to the euthyroid state, those with subclinical hyperthyroidism and TSH 0.1 to 0.44 mIU/L had an incidence rate of 59 events per 1000 person-years (P = 0.007) and a hazard ratio of 1.85 (95%CI 1.14–3.00).

Recently studies have revisited the role of TSH as a marker of disease compared to thyroid hormones: TSH is a pituitary regulatory hormone, while the effectors on the target tissues are the circulating thyroid hormones, namely T4 and especially the active T3 (section *Effects of Thyroid Hormones on Heart Rhythm and Function*). In a systematic review, Fitzgerald et al. showed that thyroid hormones were better predictors than TSH, but this was limited to observational data and no effect size was reported (27). Gammage et al. also found a cross-sectional association of AF with higher free T4 levels in participants with TSH levels within the reference range (28). A difference in free T4 of 1 pmol/L (0.08 ng/dL) was associated with an odds ratio of 1.08 (95%CI 1.03-1.14) for AF.

Based on ICD-10 codes at hospital admissions in a Danish population registry over 13 years of follow-up, Selmer et al. found that atrial flutter and AF could be predictive of subsequent subclinical hyperthyroidism (21). Among those who had a new-onset AF (mean age 66, 55% men), 3% developed subclinical hyperthyroidism after the hospitalization compared to 1% in the general population. This increased incidence was particularly seen in the first few years after the AF onset, more often with age and in women. However, these findings are most likely due to the surveillance bias because patients with atrial arrhythmia are more likely to have regular thyroid function tests. In addition, in a registry-based study, all uses of amiodarone may not have been accounted for. Finally, there is not a clear mechanistic explanation how the incidence of AF could cause subclinical hyperthyroidism.

In the context of AF, physicians and patients must take into account the association of subclinical hyperthyroidism with CHD, stroke and mortality (8), as well as the complications of AF itself (29). Since heart failure is commonly associated with AF, it is important to mention that in large observational studies, both subclinical hypothyroidism and hyperthyroidism were associated with heart failure events (30). The effect of thyroid hormones on cardiac function in patients with subclinical hypothyroidism was investigated in a nested study of the TRUST trial (185 individuals, mean age 74 years) (31). TSH decreased from a mean of 6.35 mIU/L to 3.55 mIU/L with thyroid hormone replacement while the TSH remained stable in the placebo arm (mean 5.29 mIU/L). There was no significant difference between both arms for the systolic function (LVEF: 62.7% vs. 62.5%, P = 0.72) and the diastolic function (E/e’ ratio: 10.6 vs. 10.1, P = 0.47). The cardiac function of older adults with subclinical hypothyroidism was not impacted with thyroid hormone replacement and the hypothesis that the U-shaped relationship (30) could be attenuated with therapy remains unresolved. However, this trial included a limited number of adults with TSH ≥ 10 mU/L and none with subclinical hyperthyroidism, and the association between free T4 and AF was not assessed.

### 4.1 Findings From the Thyroid Studies Collaboration

Subclinical hyperthyroidism has a prevalence of 1-2%, which limits the statistical power to examine incident events in a single cohort. To overcome this limitation, the Thyroid Studies Collaboration (TSC) sought to assess the risk of cardiovascular outcomes in SCTD by combining data from multiple cohorts. Using a common methodology, the TSC obtained data from original cohort studies and performed an individual data analysis that standardized the baseline data, the potential predictors, and the outcomes of interest. This approach was used to recalculate the risk of cardiovascular outcomes using a uniform definition of subclinical thyroid disorders and the outcomes of interest (7) (https://www.thyroid-studies.org).

**Figure 2:**
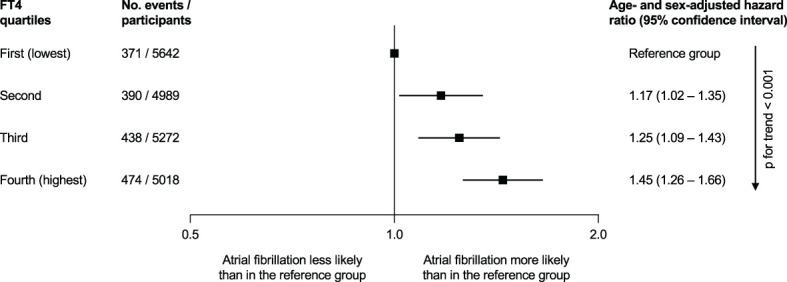
Association of free thyroxine (FT4) quartiles and the incidence of atrial fibrillation in the Thyroid Studies Collaboration, when thyroid-stimulating hormone was within the reference range (0.45 to 4.49 mIU/L) [*figure based on data by Baumgartner et al.* (24)].

Within the TSC, the risk of AF in subclinical hyperthyroidism was analyzed using participant-level data from 5 cohort studies (8). Collet et al. showed an age- and sex-adjusted HR of 1.68 (95%CI 1.16–2.43) for incident AF in subclinical hyperthyroidism. Stratified analyses showed that a lower TSH level < 0.10 mIU/L was associated with AF compared to the euthyroid reference group (HR 2.54, 95%CI 1.08-5.99). A TSH between 0.10 and 0.44 mIU/L was also associated with AF compared with the euthyroid group (HR 1.63, 95%CI 1.10-2.41). However, this analysis was limited to 5 cohort studies and the definition of AF differed among the cohorts. In a follow-up TSC analysis of 11 cohorts with 30,085 participants who were either euthyroid or had subclinical hypothyroidism (6.5%), Baumgartner et al. found no association between TSH levels in the reference range and AF, but the risk of AF increased with low-normal TSH levels in an analysis of continuous levels of TSH (32). In the analysis of free T4 in relation with AF, a higher free T4 level at baseline, even within the euthyroid range of TSH, was associated with incident AF: age- and sex-adjusted HR 1.45 (95%CI 1.26–1.66) for the highest quartile vs. the lowest quartile of free T4 (**Figure 2**).

## 5 New Tools to Detect Atrial Fibrillation in Thyroid Disorders

New data have emerged regarding the options to diagnose AF (11). An electrocardiogram (ECG) is required to formally establish the diagnosis of AF. A standard 12-lead ECG or a single-lead ECG tracing of ≥ 30 seconds showing no discernible repeating P waves and irregular RR intervals (when atrioventricular conduction is not impaired) is the diagnostic criterion of clinical AF.

The traditional tools to detect AF are multiple and include oscillometric blood pressure cuff, pulse palpation, auscultation, wearable belts for continuous recordings, long-term Holter and implantable cardiac monitors. However new devices have been developed for the screening of AF (33). Some are controlled by users, where the patient (or medical professional) initiates the measurements, such as intermittent ECG rhythm strip using a smartphone or dedicated connected device, or patient initiated photoplethysmogram on a smartphone (34). As an alternative to user-initiated recording devices, other methods use a smartwatch or wearable device: the ECG is initiated by semi-continuous photoplethysmogram and the device notifies the user in case of irregular rhythm or symptoms (35, 36). The smartphone apps have 92-98% sensitivity to detect AF with a specificity of 91%-100% compared to the 12-lead ECG (11).

The pragmatic cluster-randomized trial VITAL-AF investigated whether a one-lead wearable ECG applied in primary care can detect more AF cases compared with a traditional approach in patients older than 65 (ClinicalTrials.gov registration no NCT03515057) (37). Across 16 primary care centers in the US, 30,722 patients with no AF history were randomized to ECG screening vs. standard care. The results of this trial presented at the AHA 2020 meeting showed that the screening with the single-lead ECG was feasible but did not increase the rate of AF diagnosis compared with standard care. At 12 months, the rates of new-onset AF were 1.74% in the screening group vs. 1.60% in the control group (P = 0.33). A subgroup analysis of patients 85 and older found an absolute risk difference of 1.88% and a number needed to screen of 53. Regarding the initiation of anticoagulation, the proportion was similar in both arms (70%, P = 0.61). The authors concluded that screening for AF using a wearable ECG was feasible in primary care, but with neutral results in term of AF detection. However, this approach could potentially be more efficient in a high-risk subgroup such as older adults.

In the setting of subclinical hyperthyroidism, no study has tested whether an aggressive strategy with a regular one-lead ECG can be an approach to detect subclinical AF, especially if TSH abnormalities persist with treatment. It is also unclear how the detection of subclinical AF would finally translate into a reduction of hard clinical endpoints, such as stroke or heart failure.

## 6 Management of Subclinical Thyroid Disorders and Gaps of Knowledge

**Figure 3:**
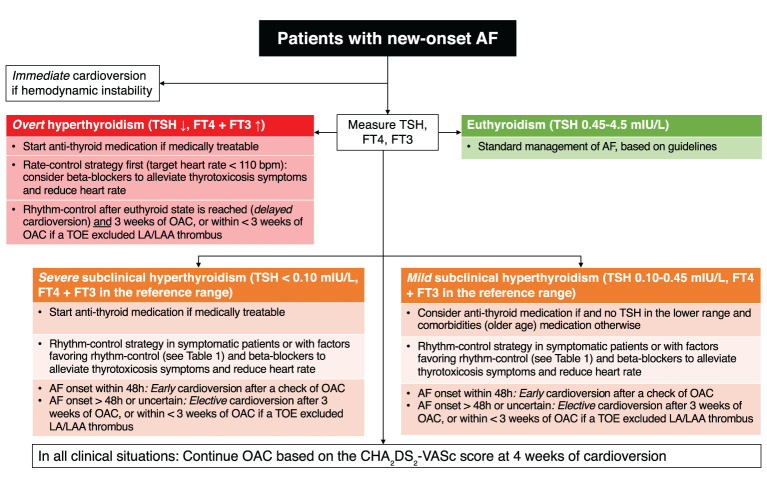
Flowchart of clinical decision making for patients with new-onset atrial fibrillation and subclinical hyperthyroidism. AF, atrial fibrillation; FT3, free triiodothyronine; FT4, free thyroxine; LA, left atrium; LAA, left atrial appendage; OAC, oral anticoagulation; TOE, trans-esophageal echocardiography; TSH, thyroid-stimulating hormone.

In the management of AF, clinical guidelines recommend the measurement of TSH in the diagnostic work-up and treatment based on the degree of thyroid abnormality (**Figure 3**). This simple screening recommendation allows clinicians to detect undiagnosed hyperthyroidism that could affect the management of AF (see section *Management of Atrial Fibrillation With Thyroid Disorder*). However, there is no data on whether treatment should be initiated in the case of newly diagnosed subclinical hyperthyroidism in a patient with preexisting AF.

Conversely, guidelines also propose to screen actively for AF in patients with known hyperthyroidism. In the case of overt hyperthyroidism, treatment is advised to avoid the symptoms and complications of hyperthyroidism (such as debilitating fatigue, anxiety, heat intolerance, arrhythmia, bone mass reduction, weight loss, thyroid storm, myxedematous coma) (38).

However, clinical recommendations differ between professional societies in the situation of subclinical hyperthyroidism. For the *European Thyroid Association* (ETA) (39), an electrocardiogram (ECG) ± 24h continuous ambulatory ECG (Holter) are recommended to assess the cardiac rhythm, and a cardiac echocardiography to assess cardiovascular morphology and function in subclinical hyperthyroidism with TSH levels < 0.1 mIU/L. Treatment could be considered in patients older than 65 years with TSH levels 0.1–0.39 mIU/L, because of their increased risk of AF, and might also be reasonable in younger (< 65 years) patients with TSH levels < 0.1 mIU/L, because of the risk of progression, especially in the presence of symptoms and/or underlying risk factors or co-morbidity. The *American Thyroid Association* (40) states that “data provide a strong argument for the treatment of subclinical hyperthyroidism in older subjects to avoid dysrhythmias and possible subsequent stroke. Whether younger patients should be treated for the same preventive indications is less clear”. The recent *French Endocrine Society* consensus statement on the management of thyroid disorders in the elderly (41) reviews the different definite therapeutic options to control hyperthyroidism based on the etiology and comorbid conditions, i.e. by surgery or radioiodine, while keeping long-course anti-thyroid medication only when these latter options are not available or not feasible. However, the authors highlight the lack of clear evidence in the case of subclinical hyperthyroidism.

Large randomized clinical trials of treatment of subclinical hyperthyroidism are needed. Of note, the randomized controlled trial on the incidence of AF after radioiodine treatment of subclinical hyperthyroidism vs. active medical surveillance conducted in France had to be terminated due to low recruitment (ClinicalTrials.gov registration no NCT00213720).

Following the TSH cut-off values of the ETA, we suggest that treatment of subclinical hyperthyroidism should be considered in patients older than 65 years with TSH < 0.4 mU/L, or in younger patients with TSH < 0.1 mU/L, due to the increased risk of AF and its complications, albeit this is still debated. The detection of AF tends to lower the threshold for initiating anti-thyroid medication. However, the decision should be balanced with the comorbidities and quality of life of the patient. In addition, the treatment of subclinical or overt hyperthyroidism should also address the cause of hyperthyroidism and not focus only on a range of laboratory values. For instance, the remission of hyperthyroidism is unlikely if it is due to a toxic adenoma. In such a case, clinical intervention with an anti-thyroid treatment (minimal invasive interventions, radioiodine or medication) would normalize TSH and thus treat the causal factor of AF.

## 7 Management of Atrial Fibrillation With Thyroid Disorder

Recommendations for AF management with SCTD are general and not always based on strong evidence. The management of patients with AF and subclinical hyperthyroidism should follow the same goals as in other patients with AF: (1) long-term prevention of AF-complications such as stroke or heart failure (2) and alleviate symptoms burden with optimal rate-control strategy or rhythm-control strategy (11). Since subclinical hyperthyroidism is a correctable cause of AF, the management should target an euthyroid state (TSH 0.45-4.49 mIU/L) to maintain cardioversion or catheter ablation and prevent recurrence of subsequent AF events. If the euthyroid state cannot be achieved for any reason, then the decision for anticoagulation should be based on available risk scores (CHA_2_DS_2_-VASc and HAS-BLED). In case of a high-risk score (CHA_2_DS_2_-VASc score ≥ 2), anticoagulation is recommended. In case of a low risk (CHA_2_DS_2_-VASc score 0-1), the persistence of subclinical hyperthyroidism and AF would be an argument for the use of anticoagulation given the possible hypercoagulable stable. The indication for the cardioversion of AF in case of persisting subclinical hyperthyroidism should be evaluated case by case. In case of invalidating symptoms, a rhythm-control strategy with an attempt of cardioversion could be indicated even in a hyperthyroid state. A regular Holter monitoring thereafter would be useful to evaluate recurrent episodes of AF.

The management of AF is based on the assessment of the 4S (Stroke risk, Symptom severity, Severity of AF burden, and Substrate Severity) (11). Hyperthyroidism could be considered as a substrate aggravating or predisposing for AF. The goal for patients with AF and subclinical hyperthyroidism is to reach the euthyroid state, although the quality of data is limited (**Figure 3**). A retrospective observational study of 163 patients with thyrotoxic AF and a mean follow-up of 34 months showed that 101 patients had spontaneous reversion and 62 patients had persisting AF (42). Those with longer duration of AF prior to thyroid function normalization were more likely to have persisting AF. In the absence of spontaneous cardioversion, pharmacological or electrical methods were described to be successful to maintain sinus rhythm (43). Beta-blockers could be used to control heart rate and heart failure, as well as in the management of thyrotoxic symptoms. In a small sample of 11 patients with hyperthyroidism, the addition of a beta-blocker improved cardiac function parameters, the control of adrenergic symptoms and quality of life (44).

Beta-blockers are indicated in the rate-control strategy of AF. Guidelines recommend the use of metoprolol, bisoprolol, carvedilol or nebivolol in patients with heart failure and reduced left ventricular ejection fraction (HFrEF) or after a myocardial infarction or as an antianginal treatment (45). When patients present both AF and HF, they can also benefit from beta-blockers (45). Propranolol is an old and nonselective beta-blocker, which is typically considered for thyrotoxicosis symptoms, including tachycardia. Compared to other beta-blockers, propranolol has a shorter elimination half-life and can be taken several times a day based on the clinical response. However, propranolol has not been trialed in patients with HFrEF or after myocardial infarction. Therefore, the use of propranolol would be indicated only in case of thyroid induced tachycardia or palpitations but without cardiac comorbidities.

The decision between the rate control and rhythm control strategies depends on the hemodynamic instability, time of onset and degree of symptoms (11). **Table 1** summarizes the criteria favoring a rhythm control strategy. In any case, hemodynamically unstable AF needs urgent cardioversion, whereas as stable AF can benefit from a cardioversion after a careful assessment of symptom onset (**Figure 3**). Those with an AF onset < 48 hours might benefit from cardioversion without starting anticoagulation, whereas for patients with an uncertain onset of symptoms, a period of 3 weeks of anticoagulation is required or alternatively a transesophageal echocardiography can be indicated to exclude the presence of thrombus in the left atrium. The correction of subclinical hyperthyroidism is indicated in AF patients with TSH < 0.10 mlU/L to eliminate a secondary cause of AF and prevent AF recurrence after cardioversion. The decision to postpone the cardioversion after reaching the euthyroid state is acceptable since the anti-thyroid medication can usually normalize T4 and T3 within their normal range in approximately 3 weeks (42).

**Table 1:** Factors favoring the rhythm control strategy, adapted from the 2020 European society of cardiology guidelines for the diagnosis and management of atrial fibrillation (AF) (11).

Younger age	
First AF episode or short history of AF onset	
Rate control target unachievable	
Tachycardia-mediated cardiomyopathy	
No or few comorbidities/heart disease	
AF precipitated by acute illness or reversible events	
AF-related symptoms	
Patient’s choice	

Another clinically relevant intersection between thyroid dysfunction and AF is amiodarone-induced thyrotoxicosis (AIT) (46, 47). Amiodarone is one of the recommended drugs for the cardioversion of AF and the maintenance of sinus rhythm, especially in case of structural heart disease and left ventricular systolic dysfunction. However, amiodarone presents several safety concerns for the long-term use, including thyroid dysfunction. Each molecule of amiodarone contains two iodine atoms, thus an estimated 3 mg of iodine is released by the liver after metabolizing 100 mg of amiodarone (46). In addition, amiodarone is lipophilic and tends to concentrate in adipose-rich tissues with a long elimination from the body (estimated half-life of 2 to 3 months (48)) and sometimes toxicity, even weeks or months after amiodarone has been discontinued, resulting in amiodarone-induced thyrotoxicosis [AIT]).

The clinical effects of amiodarone depends on the thyroid function and dietary iodine status of each individual (47). It is therefore very important to measure the thyroid function before starting the drug (46). Most patients remain euthyroid (>70%), while about 5-22% present with hypothyroidism and 2-9.6% hyperthyroidism (49). The management of AIT will depend on the thyroid function, the presence of clinical features and consist of amiodarone withdrawal, the introduction of anti-thyroid therapies and/or corticosteroids. Specialized endocrine care in case of AIT is strongly recommended due to the difficult differential diagnosis (46). In short, type I AIT leads to an increased synthesis of T4 and T3 from the excess iodine of amiodarone, whereas type II AIT is a destructive process resulting in excess release of T4 and T3 without actual synthesis. These two entities require specific management strategies and specialized endocrine care.

The choice of alternative treatments to amiodarone depends on whether a rate vs. rhythm control strategy is chosen. The classical options for rate control are beta-blockers, calcium-channel blockers and in case of left ventricular dysfunction digoxin, although this last option is not the first choice given the risk of intoxication. For rhythm control strategy, flecainide is an effective option, but needs to be used with a beta-blocker to prevent a 1:1 conduction of atrial flutter and remains contra-indicated in case of a cardiac structural disease. Other options include propafenone, vernakalant and ibutilide, but the availability of those drugs depends on the country. Of note, clinical trials testing those trials have in general excluded AF secondary to thyrotoxicosis.

SCTD are associated with increased risk of stroke, both in subclinical hypothyroidism (50) and in subclinical hyperthyroidism (51). The risk of stroke in case of hyperthyroidism-related AF does not seem to be increased compared to strokes in case of AF with euthyroidism, but there is no clear evidence to definitively conclude whether the indication for anticoagulation should differ between hyperthyroid vs. euthyroid patients (52). In 2006, the American College of Cardiology (ACC) considered hyperthyroidism as a high-risk state and recommended anticoagulation for patients in a hyperthyroid state, regardless of the CHA_2_DS_2_-VASc score (53). In contrast, the 2020 European Society of Cardiology (ESC) guidelines for the diagnosis and management of AF did not specify whether the coexistence of subclinical hyperthyroidism should change the threshold for initiating oral anticoagulation (11). Of note, the 2019 ACC guidelines did not mention hyperthyroidism either (54).

Patients with subclinical hyperthyroidism might have a hypercoagulable state due to higher concentrations of several coagulation factors in comparison to patients with euthyroidism. It is however unclear whether those biochemical abnormalities translate to a higher risk of thrombo-embolic event (55–57). Guidelines do not clearly state hyperthyroidism as a risk factor of stroke or systemic embolism in patients with AF. The use of clinical score such as CHA_2_DS_2_-VASc and HAS-BLED can help stratify the risk of thrombo-embolic vs. bleeding events in AF patients, and to guide the decision to continue anticoagulation after an episode of AF associated with hyperthyroidism. Patients with a high CHA_2_DS_2_-VASc score (≥ 2 points) or with structural abnormalities detected on the echocardiography, such as left atrium dilation or reduced ejection fraction, are factors contributing to a higher risk of complications related to AF and would probably need long-term anticoagulation even after achieving euthyroidism. In contrast, younger patients with a low CHA_2_DS_2_-VASc score (0-1 point) and normal echocardiography could discontinue anticoagulation after cardioversion and reaching euthyroid state. In any case, a follow-up monitoring with ECG or Holter cam confirm the absence of AF in the euthyroid state.

Regarding the choice of anticoagulants, vitamin K antagonists can be monitored and adapted with INR measurements in the presence of possible hypercoagulable state. The trials testing novel oral anticoagulation (NOAC) have in general excluded transient AF secondary to reversible disorder (e.g. thyrotoxicosis), and subgroups analyses according to thyroid disorder or use of thyroid medications are lacking. However, the use NOAC is recommended as first choice and no data so far reported safety concerns of NOAC in patients with thyroid disorder.

However, the distinction between subclinical and overt hyperthyroidism is not systematically well-defined in the management of AF and no range for TSH is clearly provided as a treatment target in this setting, except for the achievement of euthyroidism. The correction of FT4 and FT3 is in general detected faster in the blood while the normalization of TSH follows 6-8 weeks after treatment initiation or modification.

Since both conditions tend to occur in patients with age-related comorbidities, the CHA_2_DS_2_-VASc score should correctly predict the risk of stroke based on those comorbidities regardless of thyroid function. Of note, the initiation of oral anticoagulation is indicated in all patients three weeks prior undertaking cardioversion and needs to be pursued at least four weeks after cardioversion (11).

## 8 Conclusion

Subclinical hyperthyroidism and AF are interconnected conditions in an ageing population. The threshold for AF screening should be lower in patients with subclinical hyperthyroidism, conversely for the threshold for testing thyroid function in patients with AF. In practice, this would imply to assess regularly cardiac symptoms, undertake routine 12-lead ECG, and in case of doubt consider Holter, or wearable device monitoring. Although the evidence is limited, the same proactive approach should be considered when initiating anti-thyroid medication in patients with AF especially in case of low TSH levels (< 0.10 mlU/L). The prevention of stroke and heart failure events with oral anticoagulation remains the priority in those patients, as well as the maintenance of quality of life.

## Author Contributions

Conceptualization: BG and T-HC. Writing—original draft: BG and T-HC. Writing—review and editing: all authors. All authors contributed to the article and approved the submitted version.

## Funding

AC is supported by K24 AG042765 from the National Institute on Aging. The Thyroid Studies Collaboration is funded by grants from the Swiss National Science Foundation (SNSF 320030-172676 and 32003B-200606/1 to NR). T-HC’s research is supported by grants from the Swiss National Science Foundation (grant number PZ00P3-167826), the Leenaards Foundation, the Vontobel Foundation, the Swiss Society of Endocrinology and Diabetes, the Swiss Multiple Sclerosis Society, the Medical Board of Geneva University Hospitals, the SwissLife Jubiläumsstiftung Foundation, and the Nutrition 2000+ Foundation.

## Conflict of Interest

The authors declare that the work was conducted in the absence of any commercial or financial relationships that could be construed as a potential conflict of interest.

## Publisher’s Note

All claims expressed in this article are solely those of the authors and do not necessarily represent those of their affiliated organizations, or those of the publisher, the editors and the reviewers. Any product that may be evaluated in this article, or claim that may be made by its manufacturer, is not guaranteed or endorsed by the publisher.
